# Supplementary material for: Contrasting the development of larval and adult body plans during the evolution of biphasic lifecycles in sea urchins
Source: Development. 2024 Oct 28;151(20):dev203015. doi: 10.1242/dev.203015 (PMC11529275; doi:10.1242/dev.203015)
Supplement: Supplementary information [file develop-151-203015-s1.pdf]

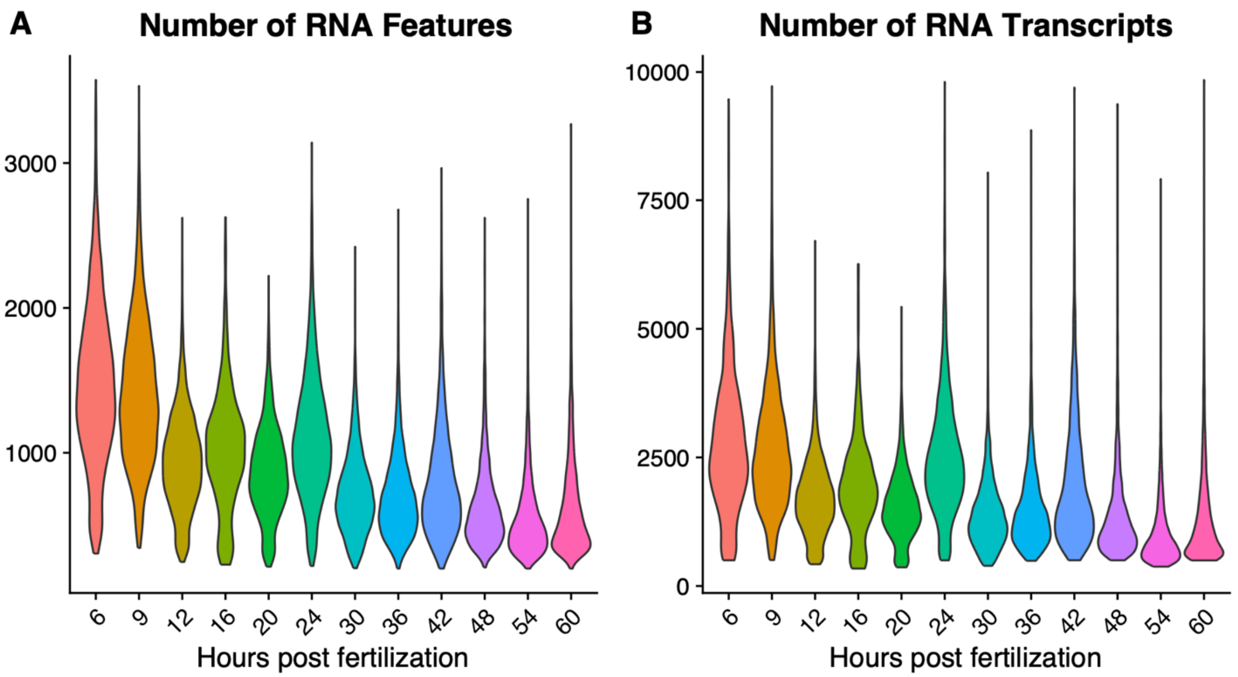

Fig. S1. Quality control plots for the *He* scRNA-seq atlas. (A) Violin plots showing the distribution of the number of RNA features (unique gene transcripts) in each cell for each sample time point. (B) Violin plots showing the distribution of the number of RNA transcripts in each cell for each sample time point.

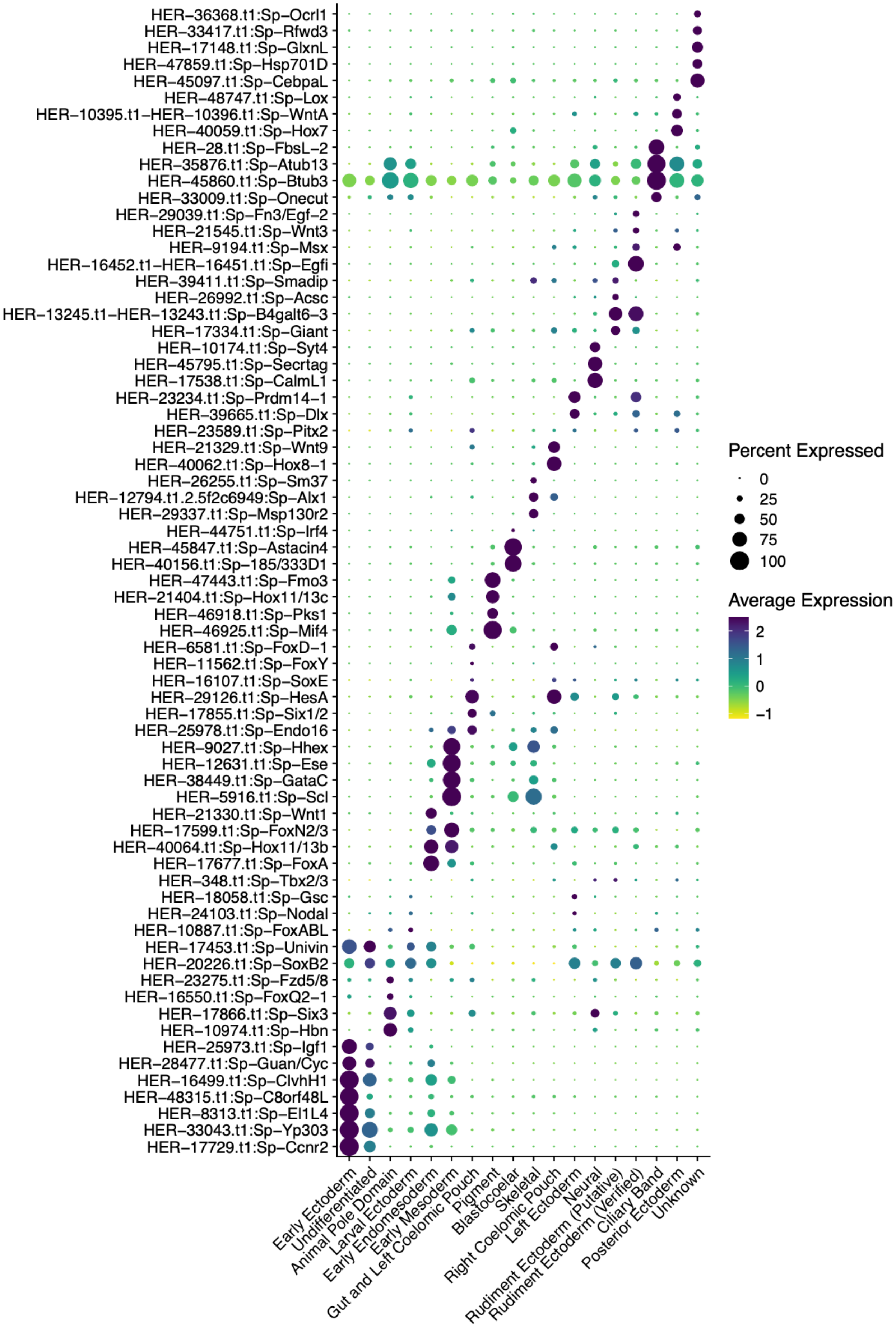

Development • Supplementary information

Fig. S2. Dot plot of cell type marker gene expression patterns. ~3-6 enriched marker genes (see Table S1) for each cell type in the scRNA-seq atlas were selected, and the average expression of the genes in each cell type was plotted.

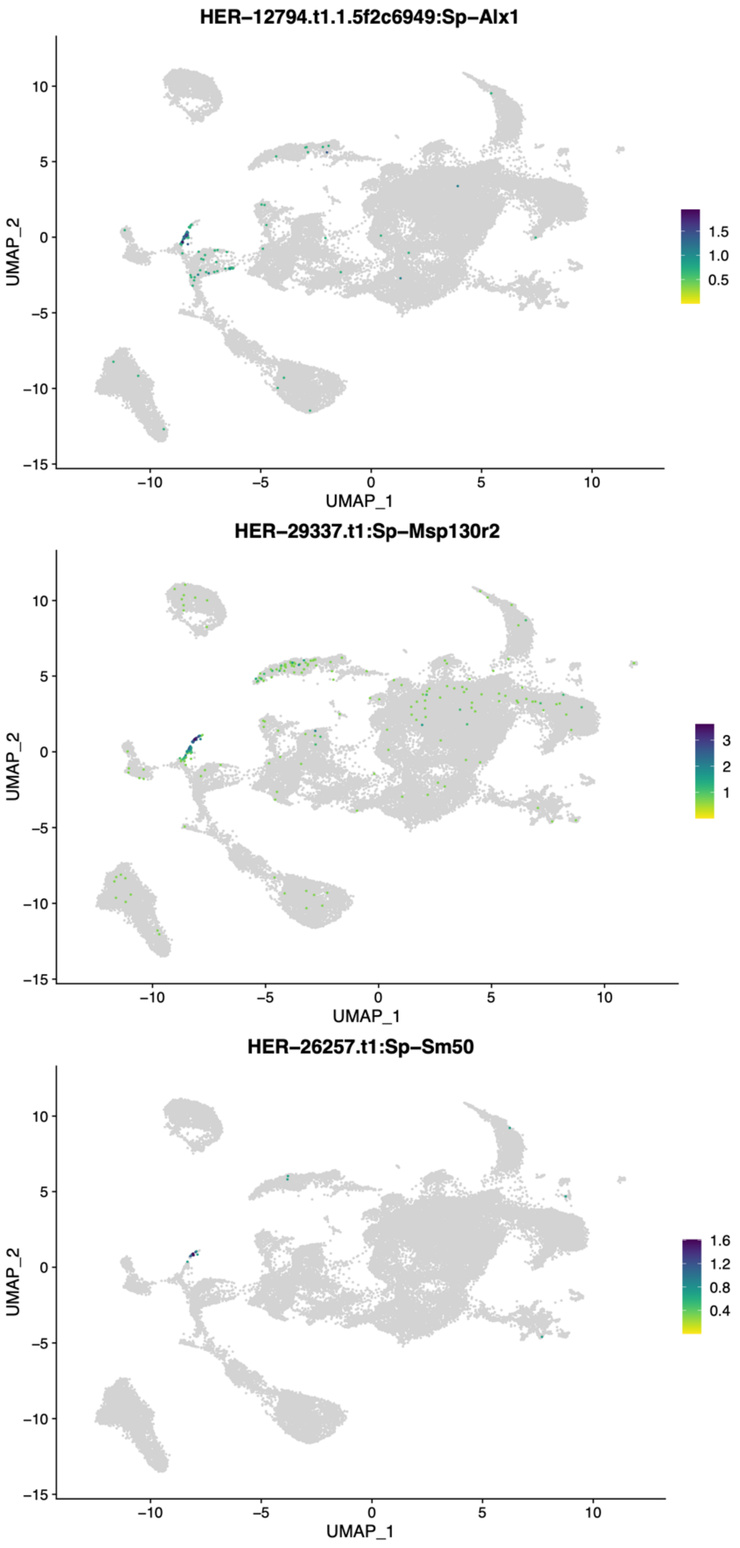

Fig. S3. Expression patterns of skeletogenic cell marker genes. These UMAPs show the expression patterns of three of the marker genes used to annotate the skeletogenic cell lineage in the *He* scRNA-seq atlas.

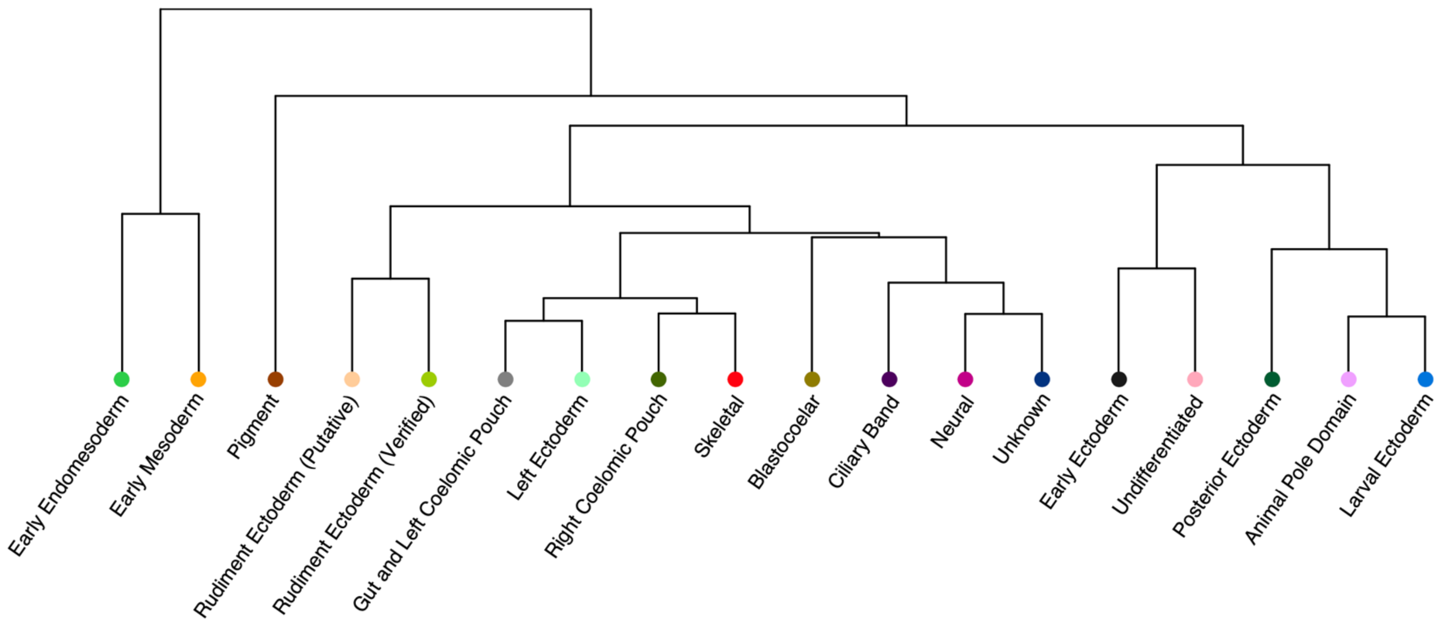

Fig. S4. Phylogenetic tree of cell types in the scRNA-seq atlas. This plot was generated using Seurat's BuildClusterTree function, which generates the phylogenetic tree using pairwise distances between the cell types in gene expression space.

Development • Supplementary information

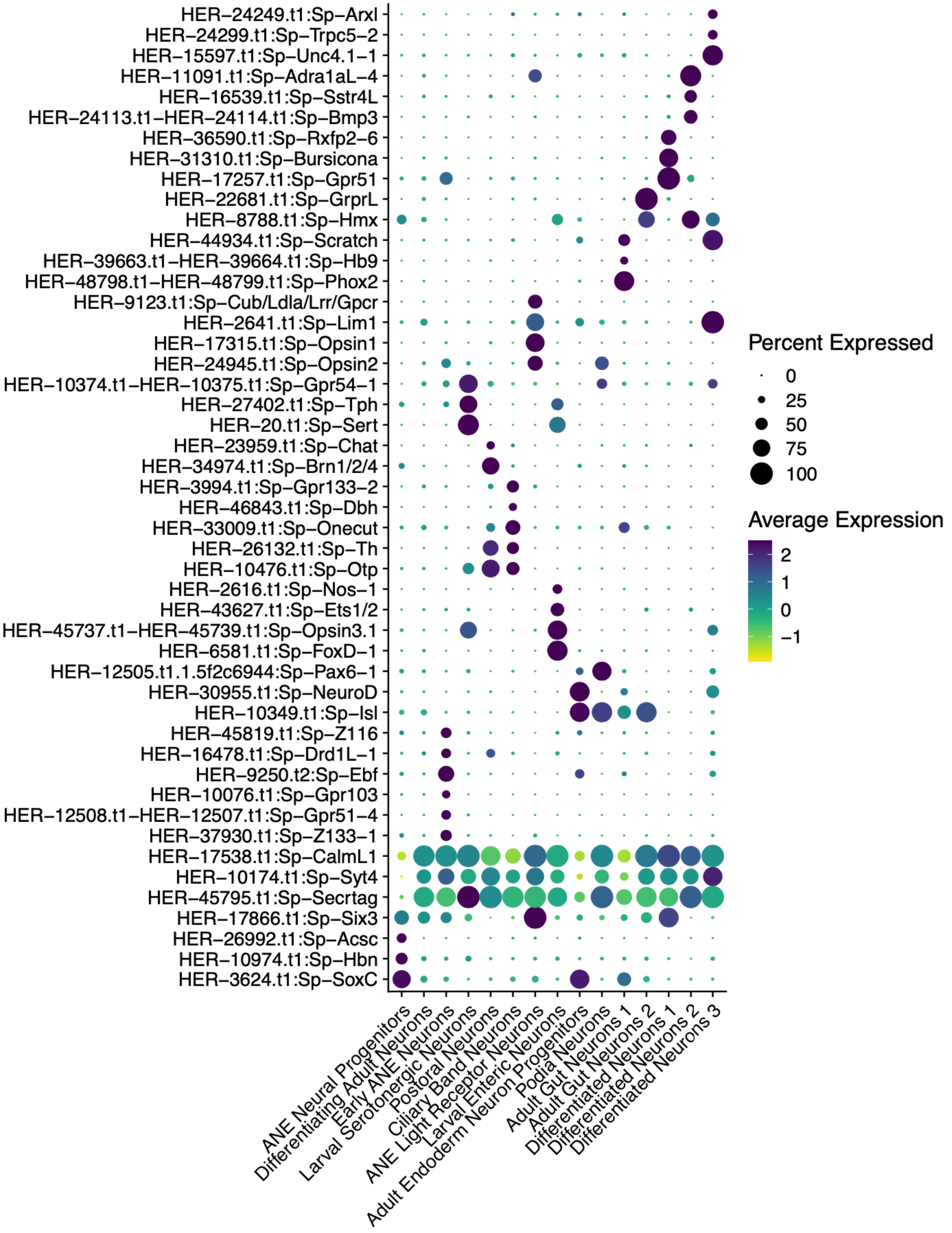

Fig. S5. Dot plot of neural cell type marker gene expression patterns. ~3-6 enriched marker genes (see Table S2) for each neural cell type in the scRNA-seq atlas were selected, and the average expression of the genes in each cell type was plotted.

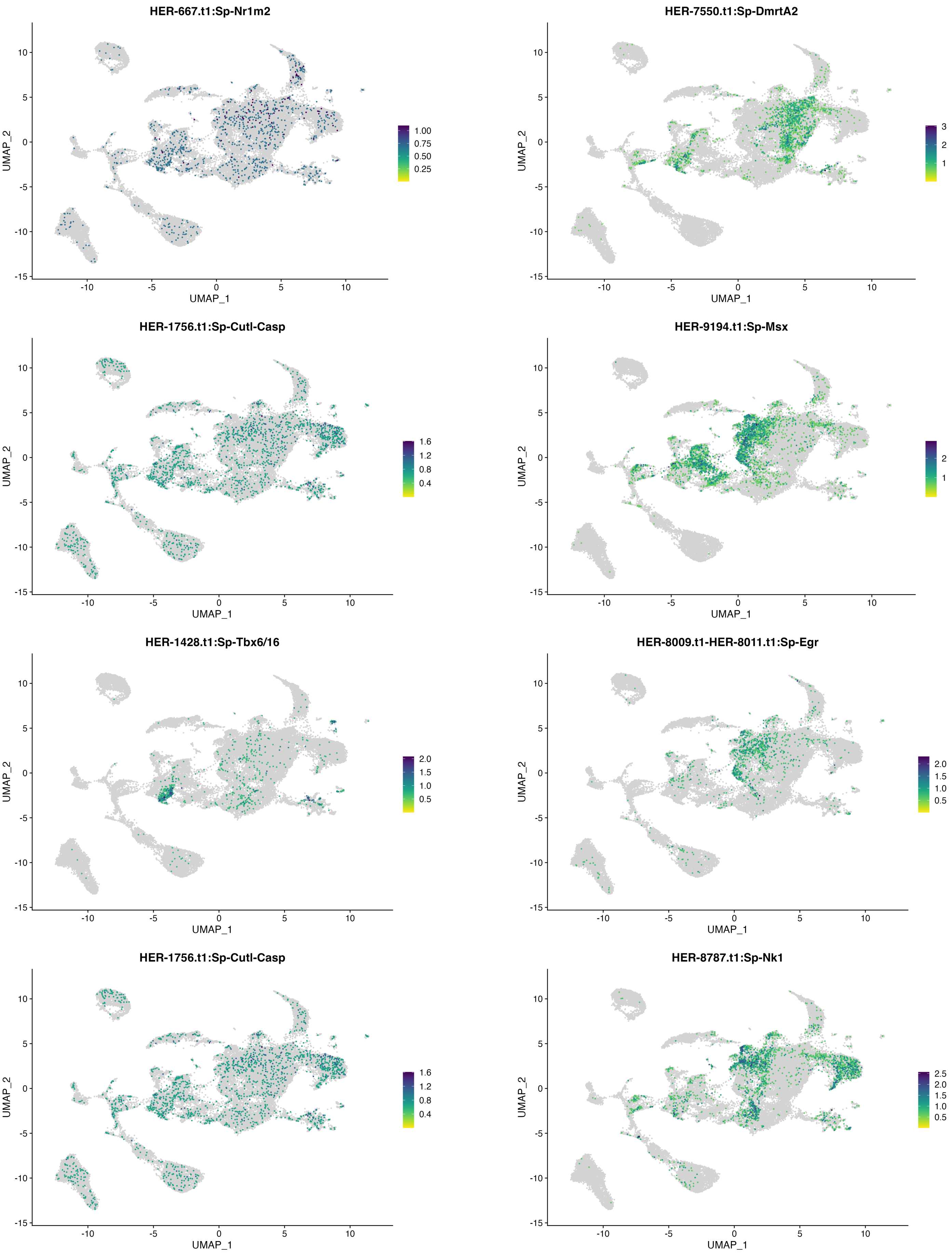
HER-667.t1:Sp-Nr1m2
UMAP_2
UMAP_1
1.00
0.75
0.50
0.25
HER-7550.t1:Sp-DmrtA2
3
2
1
HER-1756.t1:Sp-Cutl-Casp
1.6
1.2
0.8
0.4
HER-9194.t1:Sp-Msx
2
1
HER-1428.t1:Sp-Tbx6/16
2.0
1.5
1.0
0.5
HER-8009.t1-HER-8011.t1:Sp-Egr
HER-1756.t1:Sp-Cutl-Casp
HER-8787.t1:Sp-Nk1
2.5
2.0
1.5
1.0
0.5

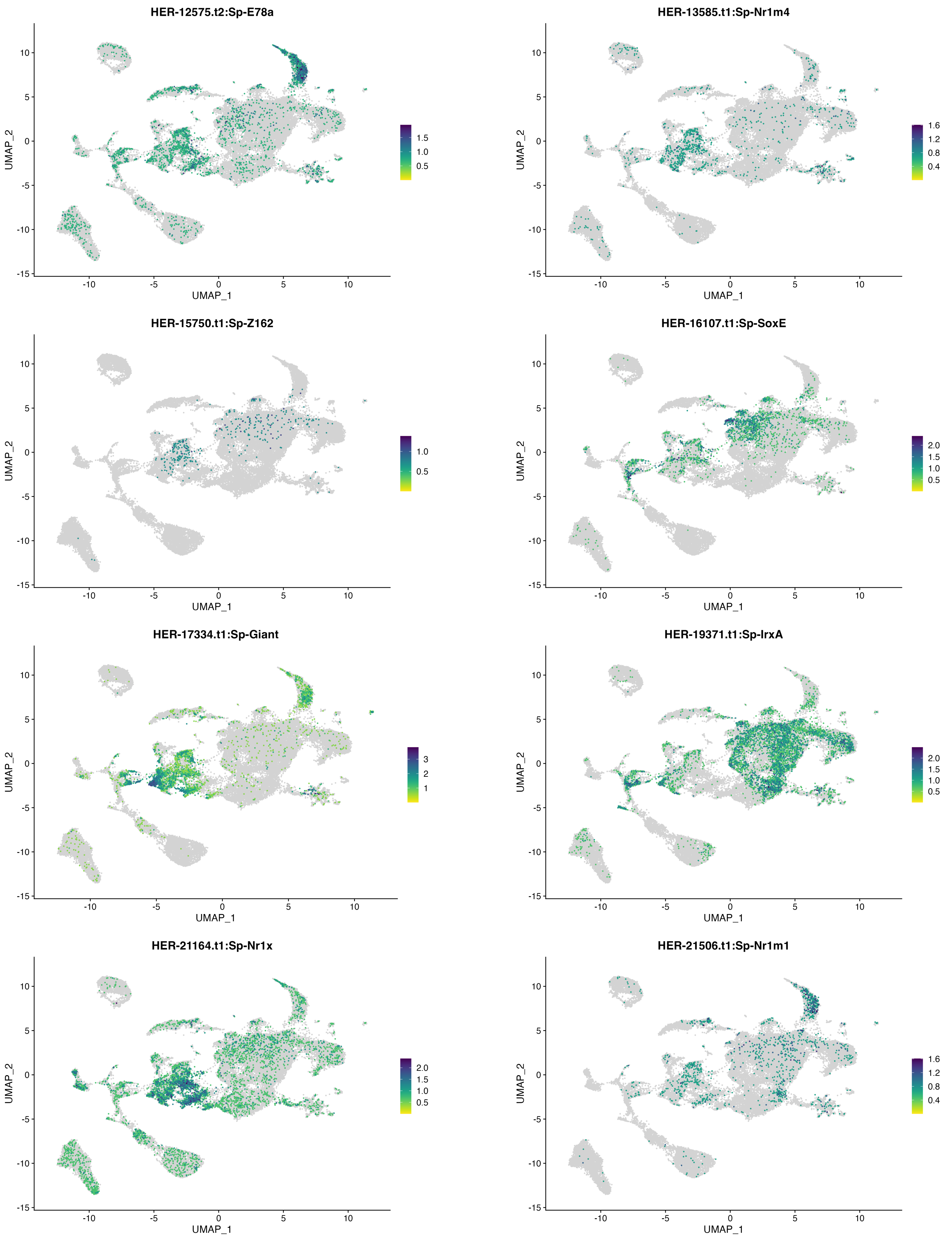

Development • Supplementary information

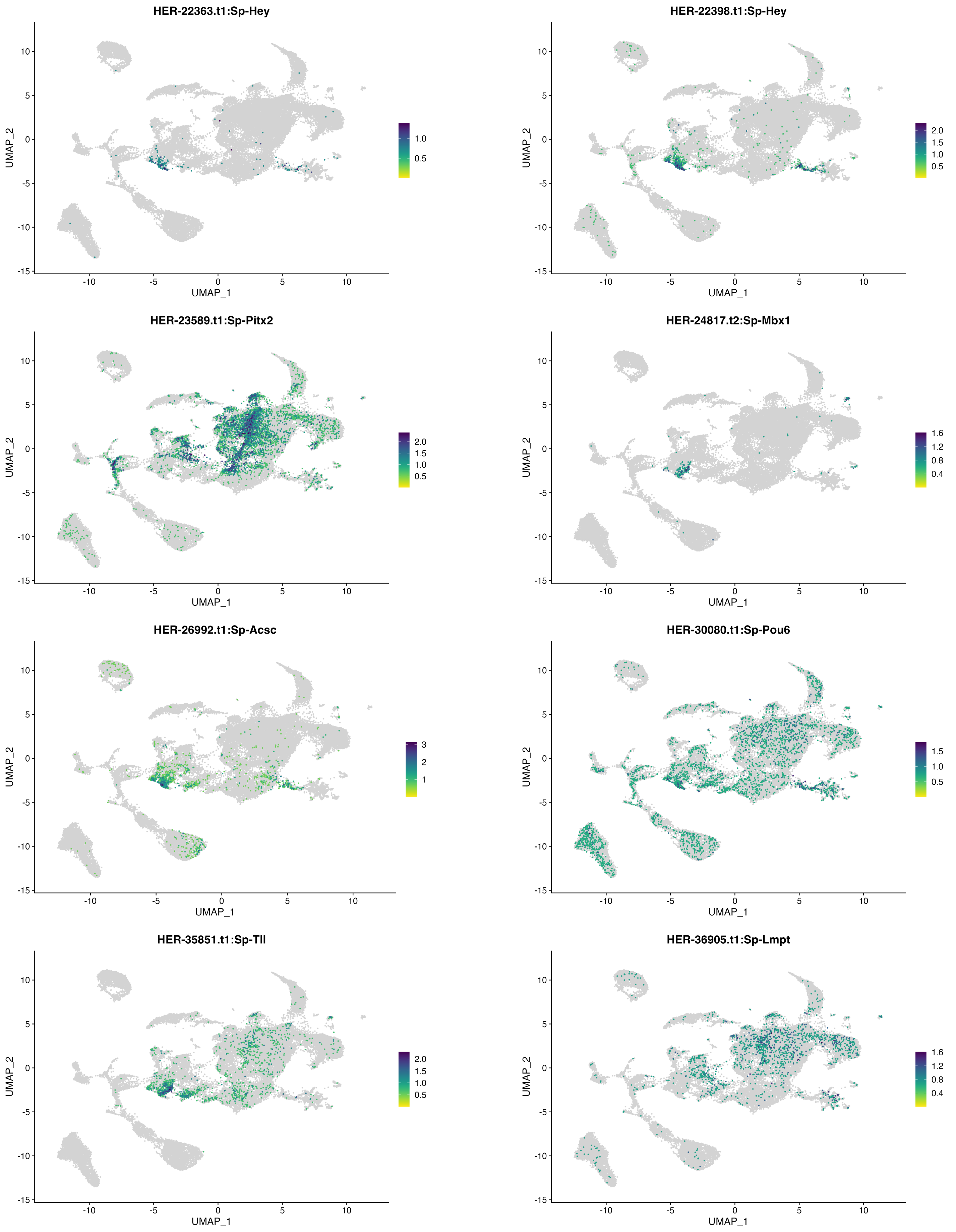

Development • Supplementary information

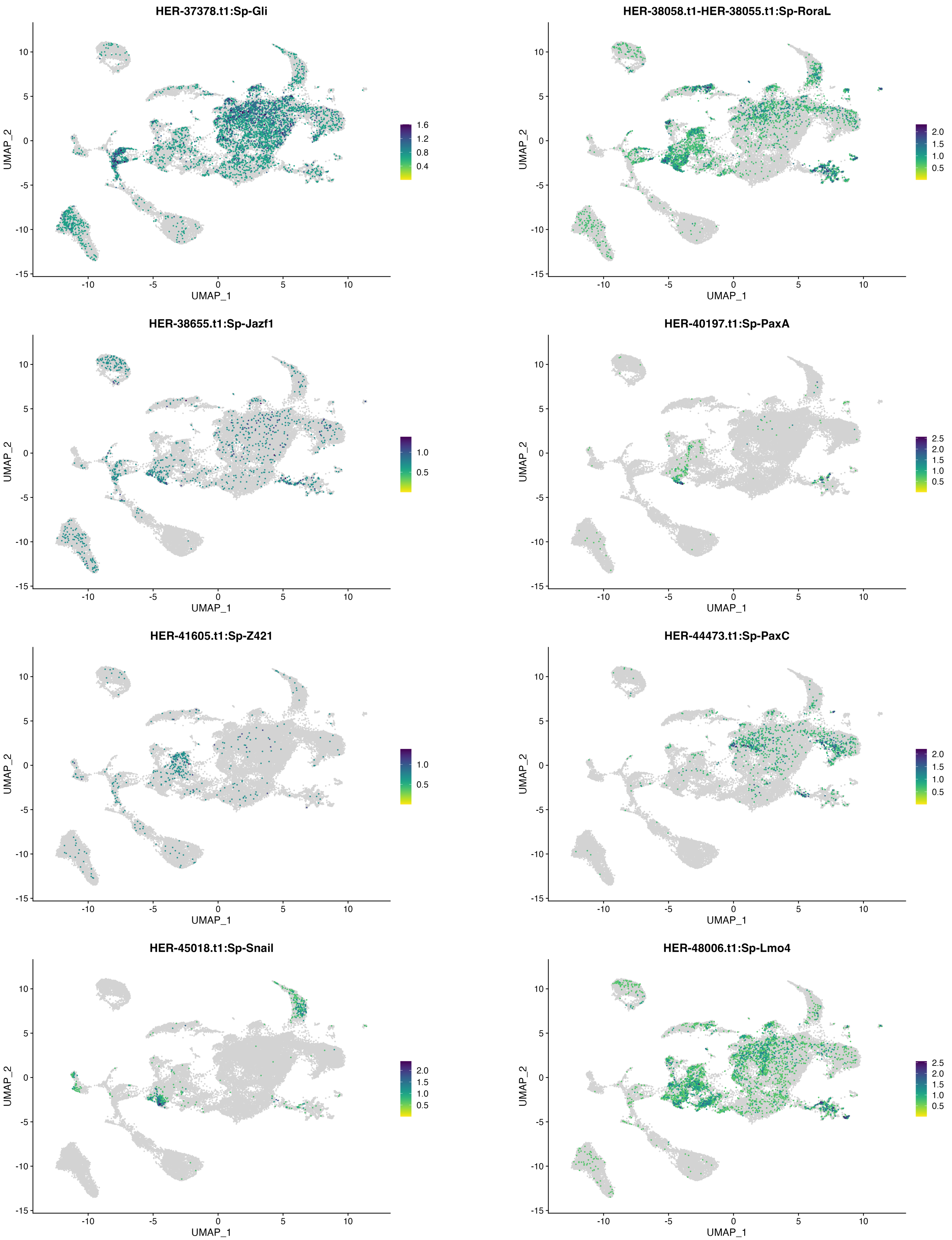

Development • Supplementary information

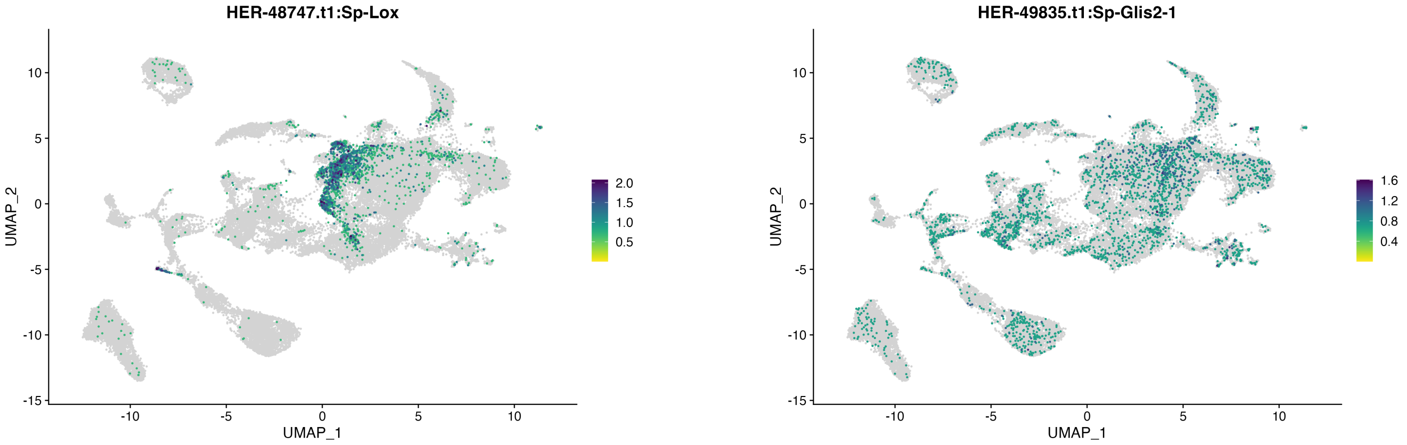

Fig. S6. Expression patterns of the candidate transcription factors for adult rudiment development. Each UMAP shows the scRNA-seq expression pattern of one of the 33 candidate transcription factors that was shown to be enriched in adult rudiment tissues (rudiment ectoderm and posterior ectoderm).

Development • Supplementary information

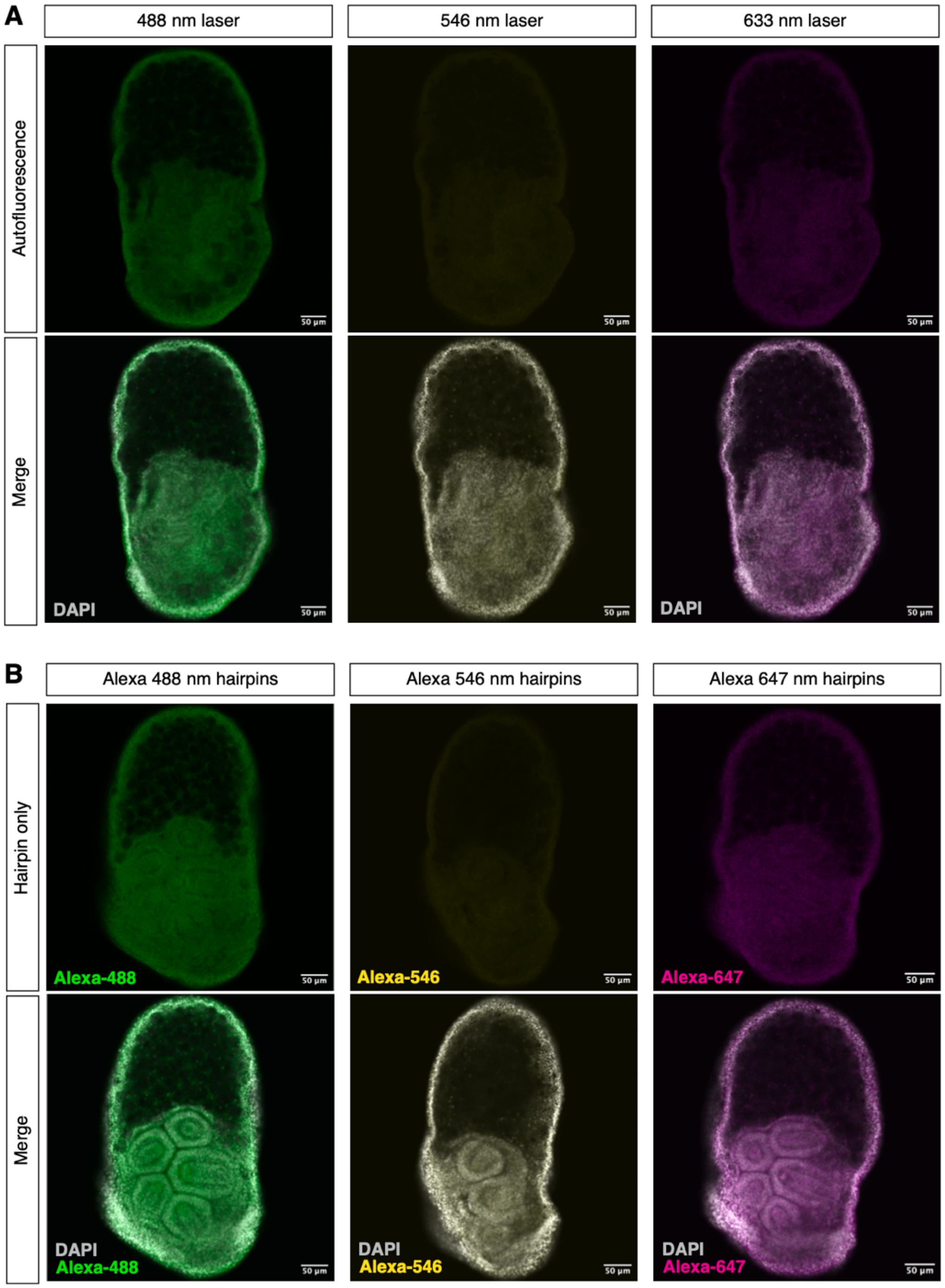

Fig. S7. Control experiments to verify HCR specificity. (A) HCR micrographs of 53 hpf *He* larvae that were incubated with no probes and no hairpins. (B) HCR micrographs of 53 hpf *He* larvae that were incubated with no probes. Neither condition resulted in localized fluorescent patterns, confirming the specificity of the probes used in this study.

Development • Supplementary information

Table S1. Cell type marker genes and references for the full scRNA-seq atlas

Available for download at
https://journals.biologists.com/dev/article-lookup/doi/10.1242/dev.203015#supplementary-data

Table S2. Neural cell type marker genes and references for the neural-only scRNA-seq atlas

Available for download at
https://journals.biologists.com/dev/article-lookup/doi/10.1242/dev.203015#supplementary-data

Table S3. List of putative transcriptional regulators of adult rudiment development

Available for download at
https://journals.biologists.com/dev/article-lookup/doi/10.1242/dev.203015#supplementary-data

Table S4. Probe sequences used in HCR experiments

Available for download at
https://journals.biologists.com/dev/article-lookup/doi/10.1242/dev.203015#supplementary-data

References (for Supplemental Data)

Allen, R. L., George, A. N., Miranda, E., Phillips, T. M., Crawford, J. M., Kiehart, D. P. and McClay, D. R. (2022). Wound repair in sea urchin larvae involves pigment cells and blastocoelar cells. *Developmental Biology*, 491, 56-65. https://doi.org/10.1016/j.ydbio.2022.08.005

Anstrom, J. A., Chin, J. E., Leaf, D. S., Parks, A. L. and Raff, R. A. (1987). Localization and Expression of Msp130, a Primary Mesenchyme Lineage-Specific Cell-Surface Protein of the Sea-Urchin Embryo. *Development*, 101, 2, 255-265. https://doi.org/10.1242/dev.101.2.255

Arenas-Mena, C., Cameron, A. R. and Davidson, E. H. (2000). Spatial expression of Hox cluster genes in the ontogeny of a sea urchin. *Development*, 127, 21, 4631-4643. https://doi.org/10.1242/dev.127.21.4631

Arenas-Mena, C., Cameron, R. A. and Davidson, E. H. (2006). Hindgut specification and cell-adhesion functions of Sphox11/13b in the endoderm of the sea urchin embryo. *Dev Growth Differ*, 48, 7, 463-472. https://doi.org/10.1111/j.1440-169x.2006.00883.x

Bellomonte, D., Di Bernardo, M., Russo, R., Caronia, G. and Spinelli, G. (1998). Highly restricted expression at the ectoderm–endoderm boundary of PIHbox 9, a sea urchin homeobox gene related to the human HB9 gene. *Mech Develop*, 74, 1-2, 185-188. https://doi.org/10.1016/S0925-4773(98)00064-1

Benson, S., Sucov, H., Stephens, L., Davidson, E. and Wilt, F. (1987). A lineage-specific gene encoding a major matrix protein of the sea urchin embryo spicule. I. Authentication of the cloned gene and its developmental expression. *Dev Biol*, 120, 2, 499-506. https://doi.org/10.1016/0012-1606(87)90253-3

Burke, R. D., Angerer, L. M., Elphick, M. R., Humphrey, G. W., Yaguchi, S., Kiyama, T., Liang, S., Mu, X., Agca, C., Klein, W. H., . . . Thorndyke, M. C. (2006). A genomic view of the sea urchin nervous system. *Dev Biol*, 300, 1, 434-460. https://doi.org/10.1016/j.ydbio.2006.08.007

Burke, R. D., Osborne, L., Wang, D., Murabe, N., Yaguchi, S. and Nakajima, Y. (2006). Neuron-specific expression of a synaptotagmin gene in the sea urchin. *J Comp Neurol*, 496, 2, 244-251. https://doi.org/10.1002/cne.20939

Buznikov, G. A., Peterson, R. E., Nikitina, L. A., Bezuglov, V. V. and Lauder, J. M. (2005). The pre-nervous serotonergic system of developing sea urchin embryos and larvae: Pharmacologic and immunocytochemical evidence. *Neurochem Res*, 30, 6-7, 825-837. https://doi.org/10.1007/s11064-005-6876-6

Calestani, C., Rast, J. P. and Davidson, E. H. (2003). Isolation of pigment cell specific genes in the sea urchin embryo by differential macroarray screening. *Development*, 130, 19, 4587-4596. https://doi.org/10.1242/dev.00647

Chen, J. H., Luo, Y. J. and Su, Y. H. (2011). The Dynamic Gene Expression Patterns of Transcription Factors Constituting the Sea Urchin Aboral Ectoderm Gene Regulatory Network. *Dev Dynam*, 240, 1, 250-260. https://doi.org/10.1002/dvdy.22514

Davidson, P. L., Guo, H., Swart, J. S., Massri, A. J., Edgar, A., Wang, L., Berrio, A., Devens, H. R., Koop, D., Cisternas, P., . . . Wray, G. A. (2022). Recent reconfiguration of an ancient developmental gene regulatory network in Heliocidaris sea urchins. *Nat Ecol Evol*, 6, 12, 1907-1920. https://doi.org/10.1038/s41559-022-01906-9

Duboc, V., Röttinger, E., Lapraz, F., Besnardeau, L. and Lepage, T. (2005). Left-right asymmetry in the sea urchin embryo is regulated by nodal signaling on the right side. *Dev Cell*, 9, 1, 147-158. https://doi.org/10.1016/j.devcel.2005.05.008

Ettensohn, C. A., Illies, M. R., Oliveri, P. and De Jong, D. L. (2003). Alx1, a member of the Cart1/Alx3/Alx4 subfamily of Paired-class homeodomain proteins, is an essential component of the gene network controlling skeletogenic fate specification in the sea urchin embryo. *Development*, 130, 13, 2917-2928. https://doi.org/10.1242/dev.00511

Ferkowicz, M. J. and Raff, R. A. (2001). Wnt gene expression in sea urchin development: Heterochronies associated with the evolution of developmental mode. *Evolution & Development*, 3, 1, 24-33. https://doi.org/10.1046/j.1525-142x.2001.00084.x

Feuda, R. and Peter, I. S. (2022). Homologous gene regulatory networks control development of apical organs and brains in Bilateria. *Sci Adv*, 8, 44. https://doi.org/10.1126/sciadv.abo2416

Garner, S., Zysk, I., Byrne, G., Kramer, M., Moller, D., Taylor, V. and Burke, R. D. (2016). Neurogenesis in sea urchin embryos and the diversity of deuterostome neurogenic mechanisms. *Development*, 143, 2, 286-297. https://doi.org/10.1242/dev.124503

Howard-Ashby, M., Materna, S. C., Brown, C. T., Chen, L., Cameron, R. A. and Davidson, E. H. (2006). Identification and characterization of homeobox transcription factor genes in Strongylocentrotus purpuratus, and their expression in embryonic development. *Developmental Biology*, 300, 1, 74-89. https://doi.org/10.1016/j.ydbio.2006.08.039

Katow, H., Suyemitsu, T., Ooka, S., Yaguchi, J., Jin-Nai, T., Kuwahara, I., Katow, T., Yaguchi, S. and Abe, H. (2010). Development of a dopaminergic system in sea urchin embryos and larvae. *J Exp Biol*, 213, 16, 2808-2819. https://doi.org/10.1242/jeb.042150

Kawasaki, T., Mitsunaga-Nakatsubo, K., Takeda, K., Akasaka, K. and Shimada, H. (1999). Lim1 related homeobox gene (HpLim1) expressed in sea urchin embryos. *Dev Growth Differ*, 41, 3, 273-282. https://doi.org/10.1046/j.1440-169X.1999.413432.x

Kenny, A. P., Oleksyn, D. W., Newman, L. A., Angerer, R. C. and Angerer, L. M. (2003). Tight regulation of SpSoxB factors is required for patterning and morphogenesis in sea urchin embryos. *Developmental Biology*, 261, 2, 412-425. https://doi.org/10.1016/S0012-1606(03)00331-2

Koop, D., Cisternas, P., Morris, V. B., Strbenac, D., Yang, J. Y., Wray, G. A. and Byrne, M. (2017). Nodal and BMP expression during the transition to pentamery in the sea urchin Heliocidaris erythrogramma: insights into patterning the enigmatic echinoderm body plan. *BMC Dev Biol*, 17, 1, 4. https://doi.org/10.1186/s12861-017-0145-1

Lapraz, F., Röttinger, E., Duboc, V., Range, R., Duloquin, L., Walton, K., Wu, S. Y., Bradham, C., Loza, M. A., Hibino, T., . . . Lepage, T. (2006). RTK and TGF-β signaling pathways genes in the sea urchin genome. *Developmental Biology*, 300, 1, 132-152. https://doi.org/10.1016/j.ydbio.2006.08.048

Lee, Y. H., Britten, R. J. and Davidson, E. H. (1999). SM37, a skeletogenic gene of the sea urchin embryo linked to the SM50 gene. *Dev Growth Differ*, 41, 3, 303-312. https://doi.org/10.1046/j.1440-169X.1999.413429.x

Materna, S. C., Swartz, S. Z. and Smith, J. (2013). Notch and Nodal control forkhead factor expression in the specification of multipotent progenitors in sea urchin. *Development*, 140, 8, 1796-1806. https://doi.org/10.1242/dev.091157

Nair, S. V., Del Valle, H., Gross, P. S., Terwilliger, D. P. and Smith, L. C. (2005). Macroarray analysis of coelomocyte gene expression in response to LPS in the sea urchin.

Identification of unexpected immune diversity in an invertebrate. *Physiol Genomics*, 22, 1, 33-47. https://doi.org/10.1152/physiolgenomics.00052.2005
Oliveri, P., Walton, K. D., Davidson, E. H. and McClay, D. R. (2006). Repression of mesodermal fate by foxa, a key endoderm regulator of the sea urchin embryo. *Development*, 133, 21, 4173-4181. https://doi.org/10.1242/dev.02577
Perillo, M., Paganos, P., Mattiello, T., Cocurullo, M., Oliveri, P. and Arnone, M. I. (2018). New Neuronal Subtypes With a "Pre-Pancreatic" Signature in the Sea Urchin. *Front Endocrinol*, 9. https://doi.org/10.3389/fendo.2018.00650
Poustka, A. J., Kühn, A., Radosavljevic, V., Wellenreuther, R., Lehrach, H. and Panopoulou, G. (2004). On the origin of the chordate central nervous system: expression of onecut in the sea urchin embryo. *Evolution & Development*, 6, 4, 227-236. https://doi.org/10.1111/j.1525-142X.2004.04028.x
Rho, H. K. and McClay, D. R. (2011). The control of foxN2/3 expression in sea urchin embryos and its function in the skeletogenic gene regulatory network. *Development*, 138, 5, 937-945. https://doi.org/10.1242/dev.058396
Rizzo, F., Fernandez-Serra, M., Squarzoni, P., Archimandritis, A. and Arnone, M. I. (2006). Identification and developmental expression of the ets gene family in the sea urchin (Strongylocentrotus purpuratus). *Developmental Biology*, 300, 1, 35-48. https://doi.org/10.1016/j.ydbio.2006.08.012
Robert, N., Lhomond, G., Schubert, M. and Croce, J. C. (2014). A comprehensive survey of wnt and frizzled expression in the sea urchin Paracentrotus lividus. *Genesis*, 52, 3, 235-250. https://doi.org/10.1002/dvg.22754
Romano, L. A. and Wray, G. A. (2006). Endo16 is required for gastrulation in the sea urchin. *Dev Growth Differ*, 48, 8, 487-497. https://doi.org/10.1111/j.1440-169x.2006.00884.x
Röttinger, E., Besnardeau, L. and Lepage, T. (2004). A Raf/MEK/ERK signaling pathway is required for development of the sea urchin embryo micromere lineage through phosphorylation of the transcription factor Ets. *Development*, 131, 9, 2233-2233. https://doi.org/10.1242/dev.01000
Slota, L. A. and McClay, D. R. (2018). Identification of neural transcription factors required for the differentiation of three neuronal subtypes in the sea urchin embryo. *Developmental Biology*, 435, 2, 138-149. https://doi.org/10.1016/j.ydbio.2017.12.015
Slota, L. A., Miranda, E. M. and McClay, D. R. (2019). Spatial and temporal patterns of gene expression during neurogenesis in the sea urchin. *Evodevo*, 10. https://doi.org/10.1186/s13227-019-0115-8
Solek, C. M., Oliveri, P., Loza-Coll, M., Schrankel, C. S., Ho, E. C. H., Wang, G. Z. and Rast, J. R. (2013). An ancient role for Gata-1/2/3 and Scl transcription factor homologs in the development of immunocytes. *Developmental Biology*, 382, 1, 280-292. https://doi.org/10.1016/j.ydbio.2013.06.019
Tu, Q., Brown, C. T., Davidson, E. H. and Oliveri, P. (2006). Sea urchin Forkhead gene family: Phylogeny and embryonic expression. *Developmental Biology*, 300, 1, 49-62. https://doi.org/10.1016/j.ydbio.2006.09.031
Ullrich-Luter, E. M., Dupont, S., Arboleda, E., Hausen, H. and Arnone, M. I. (2011). Unique system of photoreceptors in sea urchin tube feet. *Proc Natl Acad Sci U S A*, 108, 20, 8367-8372. https://doi.org/10.1073/pnas.1018495108
Wei, Z., Angerer, L. M. and Angerer, R. C. (2016). Neurogenic gene regulatory pathways in the sea urchin embryo. *Development*, 143, 2, 298-305. https://doi.org/10.1242/dev.125989

Wei, Z., Yaguchi, J., Yaguchi, S., Angerer, R. C. and Angerer, L. M. (2009). The sea urchin animal pole domain is a Six3-dependent neurogenic patterning center. *Development*, 136, 7, 1179-1189. https://doi.org/10.1242/dev.032300

Wessel, G. M., Zaydfudim, V., Hsu, Y. T. J., Laidlaw, M. and Brooks, J. M. (2000). Direct molecular interaction of a conserved yolk granule protein in sea urchins. *Dev Growth Differ*, 42, 5, 507-517. https://doi.org/10.1046/j.1440-169x.2000.00534.x

Yaguchi, J., Angerer, L. M., Inaba, K. and Yaguchi, S. (2012). Zinc finger homeobox is required for the differentiation of serotonergic neurons in the sea urchin embryo. *Developmental Biology*, 363, 1, 74-83. https://doi.org/10.1016/j.ydbio.2011.12.024

Yaguchi, J. and Yaguchi, S. (2021). Sea urchin larvae utilize light for regulating the pyloric opening. *Bmc Biology*, 19, 1. https://doi.org/10.1186/s12915-021-00999-1

Yaguchi, S. and Katow, H. (2003). Expression of tryptophan 5-hydroxylase gene during sea urchin neurogenesis and role of serotonergic nervous system in larval behavior. *J Comp Neurol*, 466, 2, 219-229. https://doi.org/10.1002/cne.10865

Yaguchi, S., Taniguchi, Y., Suzuki, H., Kamata, M. and Yaguchi, J. (2022). Planktonic sea urchin larvae change their swimming direction in response to strong photoirradiation. *Plos Genet*, 18, 2. https://doi.org/10.1371/journal.pgen.1010033

Yaguchi, S., Yaguchi, J., Angerer, R. C. and Angerer, L. M. (2008). A Wnt-FoxQ2-nodal pathway links primary and secondary axis specification in sea urchin embryos. *Dev Cell*, 14, 1, 97-107. https://doi.org/10.1016/j.devcel.2007.10.012

Development • Supplementary information
