# Supplementary material for: Contrasting the development of larval and adult body plans during the evolution of biphasic lifecycles in sea urchins
Source: Development. 2024 Oct 28;151(20):dev203015. doi: 10.1242/dev.203015 (PMC11529275; doi:10.1242/dev.203015)
Supplement: Supplementary file 1 [file develop-151-203015_review_history.pdf]

Contrasting the development of larval and adult body plans during the evolution of biphasic lifecycles in sea urchins

Brennan D. McDonald, Abdull J. Massri, Alejandro Berrio, Maria Byrne, David R. McClay and Gregory A. Wray
DOI: 10.1242/dev.203015

Editor: Karen Sears

Review timeline
Original submission: 30 April 2024
Editorial decision: 25 July 2024
First revision received: 6 September 2024
Accepted: 16 September 2024

Original submission

First decision letter

MS ID#: DEVELOP/2024/203015

MS TITLE: Contrasting the development of larval and adult body plans during the evolution of biphasic lifecycles in sea urchins

AUTHORS: Brennan D. McDonald, Abdull J. Massri, Alejandro Berrio, Maria Byrne, David R. McClay, and Gregory A. Wray

I have now received a referee report on the above manuscript, and have reached a decision. The referee's comments are appended below, or you can access them online: please go to BenchPress and click on the 'Manuscripts with Decisions' queue in the Author Area.

The overall evaluation is positive and we would like to publish a revised manuscript in Development, provided that the referee's comments can be satisfactorily addressed. Please attend to all of the reviewer's comments in your revised manuscript and detail them in your point-by-point response. If you do not agree with any of their criticisms or suggestions explain clearly why this is so. If it would be helpful, you are welcome to contact us to discuss your revision in greater detail. Please send us a point-by-point response indicating your plans for addressing the referees' comments, and we will look over this and provide further guidance.

Reviewer 1

Advance summary and potential significance to field

In this manuscript, the authors use single-cell RNA-seq data, collected from 12 distinct developmental stages, spanning both embryogenesis and larval development of the sea urchin Heliocidaris erythrogramma (He), to analyze larval to adult body plan transition in an animal with a biphasic lifecycle. During this work, the authors identified several cell clusters, some of which revealed interesting larval and adult specificities. For instance, lecithotrophic larvae seem to have lost some specific cell populations and associated regulatory genes compared to planktotrophic larvae. Lecithotrophic larvae also appear to encompass a specific set of undifferentiated cells. Furthermore, the analyses performed outlined that the development of the larval and adult body

© 2024. Published by The Company of Biologists under the terms of the Creative Commons Attribution License (https://creativecommons.org/licenses/by/4.0/).

plans of a lecithotrophic sea urchin likely relies on different sets of transcription factors. Overall, this manuscript is well-written and conveys important information that significantly contributes to a better understanding of developmental transitions in metazoans with a biphasic lifecycle.

Comments for the author

Before publication, I think the manuscript would benefit from some minor adjustments.

Throughout the manuscript, it is often difficult to understand whether the authors are referring to structures and larvae of planktotrophic species or of He. To help the reader, the authors should revise their manuscript and indicate throughout whether they are referring to He, i.e., lecithotrophic, structures and larvae, or else planktotrophic structures and larvae.

In the introduction, the authors mention that H. erythrogramma is closely related to H. tuberculata, and that they both make good models for determining how different larval developmental processes can lead to the same body plan. In the discussion, H. tuberculata is also mentioned. Yet, in the results section, the authors perform comparisons between single-cell RNA-seq data from H. erythrogramma and L. variegatus. The authors should introduce L. variegatus, explain why they chose this planktotrophic species (instead of H. tuberculata) for their comparative surveys, and mention L. variegatus in the discussion.

From the information provided in the manuscript, it is unclear why the authors decided to include in this work the 7 developmental stages that have already been published by Massri et al., 2024. According to Figure 1, the five time points from which the new unpublished scRNA-seq is derived, cover larval development from the emergence of the vestibule to that of a well-developed adult rudiment. Given that the goal of the manuscript was to compare larval and adult development, it is unclear why these five time points were not sufficient to address this question.
In the revised version of the manuscript, the authors should clarify their choice to include already analyzed and published data in the current study.

The Alx1 gene is first mentioned in the manuscript at line 186, when the authors begin reporting on gene co-expression investigations. However, the authors do not introduce this gene or explain why they chose it for their co-expression survey.
This should be clarified.

In lines 195-197, the authors conclude "these match findings from the analysis of the 6 to 30 hpf scRNA-seq dataset for He (Massri et al., 2024) and suggest that a portion of the larval skeletogenic gene regulatory network is no longer functional in He". The fact that the results of the current study are consistent with those published by Massri et al., 2024 makes sense since both studies encompass the exact same dataset, which again raises the question of why the 6 to 30 hpf time points were included in this study. Furthermore, it is unclear why the authors here conclude that a portion of the "larval" skeletogenic GRN is no longer functional in He, given that the genes they looked at are also expressed and important for skeletogenic development in embryos of planktotrophic species and might be functional in He, considering later stages. At 60 hpf, does the He adult rudiment have a skeleton? If not, the genes studied might become activated later during He development.

Also, more conceptually, how did the authors define the skeletal cluster if none of the skeletogenic GRN genes are present in this cluster? In Figure 4D, Alx1 and Scl label "a large number of mesenchymal cells surrounding the vestigial gut of early larvae". Yet, if they are indeed markers of the skeletal cluster, they should mark the larval skeleton, which, according to Figure 1, is not located where the Alx1- and Scl-labeled cells are observed. Could the author address this issue?

Regarding Hmx and Lim1 expression in larval neurons and adult rudiment tissue when does the adult nervous system form during He development? Could it be that Hmx- and Lim1-positive cells in the adult rudiment also correspond to neuronal cells? If so, these two genes would then reveal an overlap between the patterning of the nervous system in both the larva and the adult rudiment. As a matter of fact, in the discussion, the authors mention "we also noticed that several transcription factors expressed in the putative adult neurons are expressed in clusters corresponding to adult rudiment tissues".

© 2024. Published by The Company of Biologists under the terms of the Creative Commons Attribution License (https://creativecommons.org/licenses/by/4.0/).

In the paragraph starting at line 309, it is difficult to grasp whether each mention of transcription factors (TFs) refers only to the list of He TFs or else to TFs present in the embryonic GRN of planktotrophic species. And the same applies to the caption of Figure 8.

"urchins" throughout the manuscript should read "sea urchins".

First revision

Author response to reviewers' comments

Reviewer 1 Advance Summary and Potential Significance to Field:
In this manuscript, the authors use single-cell RNA-seq data, collected from 12 distinct developmental stages, spanning both embryogenesis and larval development, of the sea urchin *Heliocidaris erythrogramma* (*He*), to analyze larval to adult body plan transition in an animal with a biphasic lifecycle. During this work, the authors identified several cell clusters, some of which revealed interesting larval and adult specificities. For instance, lecithotrophic larvae seem to have lost some specific cell populations and associated regulatory genes compared to planktotrophic larvae. Lecithotrophic larvae also appear to encompass a specific set of undifferentiated cells. Furthermore, the analyses performed outlined that the development of the larval and adult body plans of a lecithotrophic sea urchin likely relies on different sets of transcription factors. Overall, this manuscript is well-written and conveys important information that significantly contributes to a better understanding of developmental transitions in metazoans with a biphasic lifecycle.

Thank you for the positive comments about the manuscript. We believe the text has thoroughly benefitted from your constructive input.

As a side note, we would like to mention that we are in the process of submitting the single cell RNA-sequencing data to a public database and will ensure that the data is available at the time of publication.

Reviewer 1 Comments for the Author:
Before publication, I think the manuscript would benefit from some minor adjustments.

Throughout the manuscript, it is often difficult to understand whether the authors are referring to structures and larvae of planktotrophic species or of *He*. To help the reader, the authors should revise their manuscript and indicate throughout whether they are referring to *He*, i.e., lecithotrophic, structures and larvae, or else planktotrophic structures and larvae.

We have tried to address any ambiguities throughout the manuscript. For example, on line **146**, we added the phrase "…in the *He* dataset…" to clarify that we were annotating the cell types of *He*, not *Lv*. Elsewhere, when we referred to the "scRNA-seq dataset", we added "*He*" as a descriptor in front to clarify which dataset and species we were referring to (such as on line **220**).

In the introduction, the authors mention that *H. erythrogramma* is closely related to *H. tuberculata*, and that they both make good models for determining how different larval developmental processes can lead to the same body plan. In the discussion, *H. tuberculata* is also mentioned. Yet, in the results section, the authors perform comparisons between single-cell RNA-seq data from *H. erythrogramma* and *L. variegatus*. The authors should introduce *L. variegatus*, explain why they chose this planktotrophic species (instead of *H. tuberculata*) for their comparative surveys, and mention *L. variegatus* in the discussion.

We thank the reviewer for pointing out this omission, and we agree that it is important to more explicitly introduce *L. variegatus* to readers. We provided an introduction to our use of *Lv* on lines **194-196**. The main reason why we chose to focus on *L. variegatus* instead of *H. tuberculata* is the presence of an existing high-quality scRNA-seq time course for the former species. While it would

© 2024. Published by The Company of Biologists under the terms of the Creative Commons Attribution License (https://creativecommons.org/licenses/by/4.0/).

be optimal to directly compare scRNA-seq data between *H. erythrogramma* and *H. tuberculata*, we chose to spend limited research funds on collecting a denser time series for *H. erythrogramma* rather than having to collect sparser time series for two separate species.
However, this is not a major limitation, as research suggests that there is limited evolutionary divergence between the developmental programs of camarodont sea urchin species with planktotrophic development, such as *H. tuberculata* and *L. variegatus*. Our published bulk RNA-seq comparative analysis of *H. tuberculata*, *H. erythrogramma*, and *L. variegatus* showed that there was strong conservation of temporal expression patterns of developmental regulatory genes in the two planktotrophic species (Israel et al., 2016, *PLOS Biology*). A few studies from the Raff lab have also found conservation of expression domains for select genes between *H. tuberculata* and *Strongylocentrotus purpuratus*, another planktotroph (e.g., Wilson et al., 2005, *Evolution & Development*; Nielsen et al., 2003, *Development Genes & Evolution*). Furthermore, several studies comparing gene expression data among planktotrophic species that diverged up to 50 million years ago again demonstrate strong conservation of developmental programs (Massri et al., 2023, *EvoDevo*; Gildor and Ben-Tabou de-Leon, 2015, *PLOS Genetics*). These results give us confidence that *L. variegatus* is a useful representative of planktotrophic development in comparative studies with *H. erythrogramma*.

From the information provided in the manuscript, it is unclear why the authors decided to include in this work the 7 developmental stages that have already been published by Massri et al., 2024. According to Figure 1, the five time points, from which the new unpublished scRNA-seq is derived, cover larval development from the emergence of the vestibule to that of a well- developed adult rudiment. Given that the goal of the manuscript was to compare larval and adult development, it is unclear why these five time points were not sufficient to address this question. In the revised version of the manuscript, the authors should clarify their choice to include already analyzed and published data in the current study.

One of our main goals with this study was to explore how the initially limited set of cell types in the embryo diversifies over the course of larval development to establish definitive adult cell types. Since previous scRNA-seq atlases in sea urchins have mostly covered stages up to the early larva, there are many outstanding questions about the developmental origins of later larval and early adult cell types. Combing the 7 early time points from Massri et al. (2024) with the 5 new late-stage time points allows us to infer cell lineage trajectories across the full development of the larva and early development of the adult body plan of *H. erythrogramma*. In addition, the analyses in Massri et al. (2024) revealed several significant differences in the specification of cell types in the *H. erythrogramma* embryo compared those in *L. variegatus*, such as in the skeletogenic cell lineage. We wanted to analyze all 12 time points together to see if these differences held across later stages. We added a brief justification to the manuscript on lines **134-135**.

The *Alx1* gene is first mentioned in the manuscript at line 186, when the authors begin reporting on gene co-expression investigations. However, the authors do not introduce this gene or explain why they chose it for their co-expression survey. This should be clarified.

Thank you for pointing out this omission. We clarified in the text on lines **178-180** that *Alx1* is a known marker for sea urchin skeletogenic cells and provided a reference to the original study that produced this finding.

In lines 195-197, the authors conclude "these match findings from the analysis of the 6 to 30 hpf scRNA-seq dataset for *He* (Massri et al., 2024) and suggest that a portion of the larval skeletogenic gene regulatory network is no longer functional in *He*". The fact that the results of the current study are consistent with those published by Massri et al., 2024 makes sense since both studies encompass the exact same dataset, which again raises the question of why the 6 to 30 hpf time points were included in this study. Furthermore, it is unclear why the authors here conclude that a portion of the "larval" skeletogenic GRN is no longer functional in *He*, given that the genes they looked at are also expressed and important for skeletogenic development in embryos of planktotrophic species and might be functional in *He*, considering later stages. At 60 hpf, does the *He* adult rudiment have a skeleton? If not, the genes studied might become activated later during *He* development.

© 2024. Published by The Company of Biologists under the terms of the Creative Commons Attribution License (https://creativecommons.org/licenses/by/4.0/).

To address your first concern about the repetition of results, we want to be a bit more precise about the extent of the analyses in each of the papers. In Massri et al. (2024), we noticed that there was little co-expression between *Alx1* and *FoxB* in the skeletogenic cells of *He* and also found that there was little *Tbr* expression in the skeletogenic population. As the aim of that paper was limited to regulatory interactions in the early embryo and larva, this finding was not pursued further. We decided to follow up on this interesting observation in the current study by looking at two additional genes, *FoxO* and *Tel*, which similarly showed a loss of co-expression. This pattern held with the addition of the 5 later time points, indicating a substantive regulatory change in the skeletogenic cells of *He* across larval development. We clarified how the current study built on the initial observations of Massri et al. (2024) on lines **200-202**.

In response to your second concern about our conclusion that a portion of the larval skeletogenic GRN is no longer functional in *He*, we agree that our language could be more precise. Instead of saying that a portion of the network is no longer functional, we say that “a subset of the interactions in the larval skeletogenic gene regulatory network has been lost in *He*” (see lines **200-202**). To clarify how we arrived at this finding, we would like to note that there are several transcription factors that play a role in specifying the skeletogenic cells of sea urchin larvae (for a comprehensive study, see c). The Gao and Davidson (2008, *PNAS*) study established that 4 of these genes--*Tbr*, *Tel*, *FoxO*, and *FoxB*--are expressed only in cells that will give rise to the larval skeleton, but not in cells that will give rise to the adult skeleton. In our study, we found that these 4 genes show reduced expression in all skeletogenic cells in *He*, though they continue to be expressed in other cell populations. This suggests that these genes no longer play a role in specifying the skeletogenic cell lineage in *He*. Our interpretation of this finding is that skeletogenic cells in *He* larvae no longer display the characteristics of the cells that build the larval skeleton in planktotrophic sea urchins. Instead, our second set of analyses (showing co-expression between *Alx1* and *Scl*, *GataC*, and *Ese*) suggests that skeletogenic cells in *He* larvae are more similar to the cells that produce the adult skeleton in planktotrophic sea urchins. For a more detailed analysis of how specific components of the ancestral sea urchin developmental gene regulatory network have been altered in *He*, please see Massri et al. (2024).

Evidence indicates that the adult skeleton has begun to form in *He* by 60 hpf. Phase-contrast micrographs of 51 hpf *He* larvae from a previous study show the presence of some skeletal spicules that will go on to form the adult skeletal test (see Fig. 3A-B in Klueg et al. (1997), *Developmental Biology*).

Also, more conceptually, how did the authors define the skeletal cluster if none of the skeletogenic GRN genes are present in this cluster? In Figure 4D, *Alx1* and *Scl* label "a large number of mesenchymal cells surrounding the vestigial gut of early larvae". Yet, if they are indeed markers of the skeletal cluster, they should mark the larval skeleton, which, according to Figure 1, is not located where the *Alx1*- and *Scl*-labeled cells are observed. Could the author address this issue?

We thank the reviewer for raising this important point, as our language about the definition of the skeletogenic cell cluster was unclear. We defined the skeletal cell cluster as the cells that expressed the genes *Alx1*, *Msp130*, *Sm37*, and *Sm50*, which are known markers of skeletogenic cells in sea urchins (see Table S1 for literature references) and have been used to annotate skeletogenic cells in prior sea urchin scRNA-seq atlases (Massri et al., 2021, *Development*; Paganos et al., 2021, *Elife*). These genes showed very localized expression patterns in the putative skeletogenic cell cluster, giving us high confidence in our cell type annotation **(see Fig. S3)**. In our revisions, we made the definition of this cluster clearer on lines **178-180**.

One of our main conclusions about SKCs in *He* was that a subset of the genes (*Tbr*, *Tel*, *FoxO*, and *FoxB*) in the GRN of larval skeletogenic cells are no longer expressed in the skeletogenic cells of *He*. However, the expression in these same cells of *Msp130* and *Sm37*, which are downstream structural and metabolic genes of terminally differentiated skeletogenic cells, provides strong evidence that skeletogenic cells are still present in *He* larvae. We have now added a supplemental figure **(Fig. S3)** with UMAPs showing the expression territories of SKC marker genes. Regarding the placement of the skeleton in the Fig. 1 diagram, only the skeletal spicules from the remnant larval skeleton are depicted, while the developing adult skeleton is not shown. We agree that this was confusing and **removed the depiction of the skeleton from the diagram**, since the main

© 2024. Published by The Company of Biologists under the terms of the Creative Commons Attribution License (https://creativecommons.org/licenses/by/4.0/).

purpose of the figure was to show rudiment development. While *Alx1* expressing cells are widespread in the micrograph in Fig. 4D, likely only a subset of these is producing the small larval skeleton, while the rest are likely cells that will produce the adult skeleton but have not yet reached their terminally differentiated fates.

Regarding *Hmx* and *Lim1* expression in larval neurons and adult rudiment tissue, when does the adult nervous system form during *He* development? Could it be that *Hmx*- and *Lim1*-positive cells in the adult rudiment also correspond to neuronal cells? If so, these two genes would then reveal an overlap between the patterning of the nervous system in both the larva and the adult rudiment. As a matter of fact, in the discussion, the authors mention "we also noticed that several transcription factors expressed in the putative adult neurons are expressed in clusters corresponding to adult rudiment tissues".

While there is no published atlas of adult nervous system development in *He*, Ferkowicz and Raff (2008, *Evolution & Development*) mention that radial nerves are present in the developing adult tube feet at least as early as 44 hpf. Byrne et al. (2018, *Developmental Dynamics*) also explores adult nervous system development in *He* larvae. The *in situ* expression pattern of *Pax6* in 40 hpf *He* larvae in this paper aligns with our identification of *Pax6*-expressing neural cells in our scRNA-seq dataset as potential adult neurons. These studies suggest that our scRNA-seq dataset likely captures developing adult neurons from multiple developmental stages.

Based on HCRs, it appears that *Hmx* and *Lim1* positive cells are part of the larval nervous system as well as ectodermal tissue in the adult rudiment. In the scRNA-seq data, *Hmx* and *Lim1* positive cells are found in clusters corresponding to neurons as well as vestibular ectoderm. We agree that the expression domain in the rudiment could include neurons as well as other ectodermal cells, thereby indicating overlap between the patterning of the nervous system in both the larva and adult rudiment. The paragraph starting at line **270** originally ended with the sentence, "It appears that there is overlap between the patterning of the nervous system and the adult rudiment in *He* larvae", to which we now added the clause, "with similar genes potentially controlling neurogenesis in both larvae and adults". This partially aligns with a previous study that analyzed bulk RNA-seq samples from before and after *He* metamorphosis. Wygoda et al. (2014, *Genome Biology and Evolution*) found that many neural genes were expressed in both pre- and post-metamorphic samples, though there were also several neural genes that were expressed only in post-metamorphic stages. In the current study, scRNA-seq allowed us to specifically distinguish between potential larval and adult neural populations and identify the regulatory genes that were expressed in each.

In the paragraph starting at line 309, it is difficult to grasp whether each mention of transcription factors (TFs) refers only to the list of *He* TFs or else to TFs present in the embryonic GRN of planktotrophic species. And the same applies to the caption of Figure 8.

In the paragraph the reviewer is referring to, all mentions of the abbreviation "TFs" refer to the list of transcription factors in the *He* genome that we curated for this paper. We modified the language at several locations in this paragraph to make it clear that "TFs" refers to this *He*-specific list, while "transcription factors" is used to refer to these genes in general across multiple species.

"urchins" throughout the manuscript should read "sea urchins".

Thank you for this suggestion. We have fixed this issue throughout the manuscript.

<u>Second decision letter</u>

MS ID#: DEVELOP/2024/203015

MS TITLE: Contrasting the development of larval and adult body plans during the evolution of biphasic lifecycles in sea urchins

© 2024. Published by The Company of Biologists under the terms of the Creative Commons Attribution License (https://creativecommons.org/licenses/by/4.0/).

AUTHORS: Brennan D. McDonald, Abdull J. Massri, Alejandro Berrio, Maria Byrne, David R. McClay, and Gregory A. Wray

I am happy to tell you that your manuscript has been accepted for publication in Development, pending our standard publication integrity checks.

© 2024. Published by The Company of Biologists under the terms of the Creative Commons Attribution License (https://creativecommons.org/licenses/by/4.0/).
